# Enhanced anti-melanoma efficacy through a combination of the armed oncolytic adenovirus ZD55-IL-24 and immune checkpoint blockade in B16-bearing immunocompetent mouse model

**DOI:** 10.1007/s00262-021-02946-z

**Published:** 2021-04-26

**Authors:** Hai-Jun Hu, Xiu Liang, Hai-Lang Li, Huai-Yuan Wang, Jin-Fa Gu, Lan-Ying Sun, Jing Xiao, Jin-Qing Hu, Ai-Min Ni, Xin-Yuan Liu

**Affiliations:** 1 State Key Laboratory of Cell Biology, Shanghai Institute of Biochemistry and Cell Biology, Center for Excellence in Molecular Cell Science, Chinese Academy of Sciences, Shanghai, 200031 China; 2 University of Chinese Academy of Sciences, Beijing, 100049 China; 3 School of Life Sciences and Technology, Tongji University, Shanghai, 200092 China; 4 Department of Pharmacy, Xiamen Medical College, Xiamen, 361023 China

**Keywords:** Melanoma, Oncolytic virus, PD-1 blockade, Combination therapy, Tumor immune infiltration, Tumor immune recognition

## Abstract

Although the recent treatment in melanoma through the use of anti-PD-1 immunotherapy is successful, the efficacy of this approach remains to be improved. Here, we explore the feasibility of combination strategy with the armed oncolytic adenovirus ZD55-IL-24 and PD-1 blockade. We find that combination therapy with localized ZD55-IL-24 and systemic PD-1 blockade leads to synergistic inhibition of both local and distant established tumors in B16-bearing immunocompetent mouse model. Our further mechanism investigation reveals that synergistic therapeutic effect is associated with marked promotion of tumor immune infiltration and recognition in both local and distant tumors as well as spleens. PD-1 blockade has no obvious effect on promotion of tumor immune infiltration and recognition. Localized therapy with ZD55-IL-24, however, can help PD-1 blockade to overcome the limitation of relatively low tumor immune infiltration and recognition. This study provides a rationale for investigation of such combination therapy in the clinic.

**Supplementary information:**

The online version contains supplementary material available at 10.1007/s00262-021-02946-z.

## Introduction

Melanoma, the most aggressive type of skin cancer, has a poor prognosis, with a median overall survival of 8–10 months and a 5-year survival rate of 20% [1]. Even with early diagnosis, melanoma still exhibits a poor prognosis because of its rapid proliferation, and therapy remains challenging for physicians [2]. In the past several years, five breakthrough anti-melanoma agents have been approved by the US Food and Drug Administration (FDA). These agents include small-molecule inhibitors of BRAF and MEK, immunotherapeutic antibodies directed at cytotoxic T lymphocyte-associated protein 4 (CTLA-4) and programmed cell death protein 1 (PD-1), and the modified oncolytic herpes virus T-VEC [3]. Recent studies suggest that anti-PD-1 therapy showed the best outcomes among the current melanoma therapeutic agents and, therefore, it is considered as the current best anti-melanoma drug [4]. However, the dramatic responses of anti-PD-1 therapy are confined to a minority of patients, and most treated patients still succumb to progressive disease [5]. Therefore, although the current treatments for melanoma have been successful, to cure or make melanoma manageable chronic, more effective drugs or therapeutic strategies are still needed.

To address the above issue, there is currently much enthusiasm for combining anti-PD-1 therapy with other cancer treatment modalities [6–8]. CTLA-4 and PD-1 inhibit antitumor immune responses by different mechanisms; consistent with preclinical studies that demonstrated synergistic effects of combined anti-CTLA-4 with anti-PD-1 therapy, a phase III clinical trials demonstrated that combination therapies led to improved responses in patients with metastatic melanoma [9]. Another compelling strategy is combination of immune checkpoint inhibitors with T-VEC. Recent studies indicated that the efficacy of anti-PD-1 antibodies was largely confined to patients with tumors that have robust baseline CD8^+^ T cell infiltrates [10], and the modified oncolytic herpes virus T-VEC could enhance the immunogenicity of melanomas for which baseline immune recognition was lacking or not robust [3]. Preliminary reports have described high response rates in patients with advanced-stage melanoma treated using the combination of anti-PD-1 therapy and T-VEC therapy [3, 11]. Thereby, it is reasonable to suggest that a combination of complementary therapeutic strategies has a better chance of success.

Oncolytic viruses are self-amplifying cancer biotherapeutics that destroy cancerous tissues without causing harm to normal tissues [12]. ZD55 is an E1B 55-kDa gene-deleted adenovirus that is similar to the famous oncolytic adenovirus ONYX-015, but with the marked difference of a cloning site to insert foreign genes [13]. Another two differences between ZD55 and other oncolytic adenoviruses are that ZD55 is an adenovirus type 5 homozygote and has an intact E3 region, whereas the most of other oncolytic adenoviruses either are adenovirus chimeras (e.g., ONYX-015 is a type 2/5 chimera and Colo-Ad1 is a type 11/3 chimera) [12–14] or have major deletions within the E3 region (e.g., ONYX-015, H101 and CG0070) [12, 14, 15]. Based on our tumor-targeting replicative viral vector ZD55, we constructed ZD55-IL-24 by inserting the exogenous antitumor gene mda-7/interleukin-24 (IL-24) gene into the cloning site of ZD55 [16]. ZD55-IL-24 was one of the most effective armed oncolytic viruses we have found yet [17]. Our previous results have shown that ZD55-IL-24 was superior to ONYX-015 and the replication-defective adenovirus carrying the IL-24 gene [16, 18–20]. In this study, we further investigated whether ZD55-IL-24 therapy in combination with PD-1 immune checkpoint inhibition could facilitate primary tumor as well as metastatic lesions rejection in B16-bearing immunocompetent mouse model.

## Materials and methods

### Antibodies

Therapeutic anti-PD-1 (BE0146) and isotype control antibody (BE0089) were produced by BioXcell. Antibodies used for flow cytometry were purchased from eBioscience (Anti-mouse CD3ε FITC, CD11b eFluor 660) and Biolegend (Anti-mouse CD8a Alexa Fluor® 647, NK 1.1 Alexa Fluor® 647, Ly-6G/Ly-6C (Gr-1) Alexa Fluor® 488, CD19 FITC, CD45R/B220 Alexa Fluor® 647, F4/80 Alexa Fluor® 488, CD206 Alexa Fluor® 647, FOXP3 Alexa Fluor® 488, CD4 Alexa Fluor® 647, CD11c Alexa Fluor® 488, I-A/I-E Alexa Fluor® 647, CD16/32).

### Cells

All the cell lines used in this study were obtained from the Cell Bank of the Type Culture Collection of the Chinese Academy of Sciences (Shanghai, China). HEK293 cell was a human embryonic kidney cell line, transformed with Ad5 E1. B16 was the murine melanoma cell line. The HEK293 cell line was cultured in DMEM (Gibco, 11995-073), and B16 cell line was cultured in RPMI-1640 (Gibco, 11875-093). Media were supplemented with 10% FBS (Gibco, 10270-106), 50 U/mL penicillin and 50 μg/mL streptomycin. All cell lines were maintained at 37 °C with 5% CO_2_ and tested to ensure that they were free of mycoplasma contamination [21].

### Adenoviruses

The recombinant adenoviruses ZD55-IL-24 used in this study have been previously described [13, 16]. Briefly, ZD55 is a conditionally replicating adenovirus type 5 with E1B (55-kDa)-deleted and ZD55-IL-24 is a foreign human IL-24 gene-inserted ZD55 expressing IL-24. ZD55-IL-24 was propagated in HEK293 cells and purified by CsCl equilibrium centrifugation. Virus titers were determined by TCID50 assay using HEK293 cells and converted to plaque-forming units (PFU). The titer of ZD55-IL-24 was adjusted to 1.5 × 10^10^ PFU/mL and was administered intratumorally at 50 μL/dose.

### Mice

All mice were obtained from Shanghai Laboratory Animal Center, Chinese Academy of Sciences (SLAC) (Shanghai, China) and maintained at ≤ 5 mice per cage under specific pathogen-free conditions in the Animal Care Facility of Shanghai Institute of Biochemistry and Cell Biology, Chinese Academy of Sciences. Mice were provided with water and rodent chow, and maintained under a regular 12 h/12 h light/dark schedule at a constant room temperature (22 ± 2 °C). The responsible veterinarian was in charge of the diagnosis, treatment, and control of diseases in the animal facilities. All animal procedures were performed in strict accordance with the institutional guidelines and were approved by the Institutional Animal Care and Use Committee of Shanghai Institute of Biochemistry and Cell Biology, Chinese Academy of Sciences. Special attention was taken to determine the humane end points and to decide whether the mice should be euthanized in order to avoid further suffering. According to the 3Rs principle, experiments were carefully designed to minimize the use of mice and to obtain maximum amount of data. Animal experiments were reported in accordance with the ARRIVE guidelines [22].

### Flow cytometry

Cells isolated from tumors or spleens were processed for surface labeling with appropriate antibodies. Propidium iodide was used to distinguish the live cells. FOXP3 was stained using the True-Nuclear™ Transcription Factor Buffer Set (424401) from Biolegend. Data were acquired using the Beckman CytoFLEX Flow Cytometer and analyzed using FlowJo software.

### Local tumor therapy experiment

**Fig. 1:**
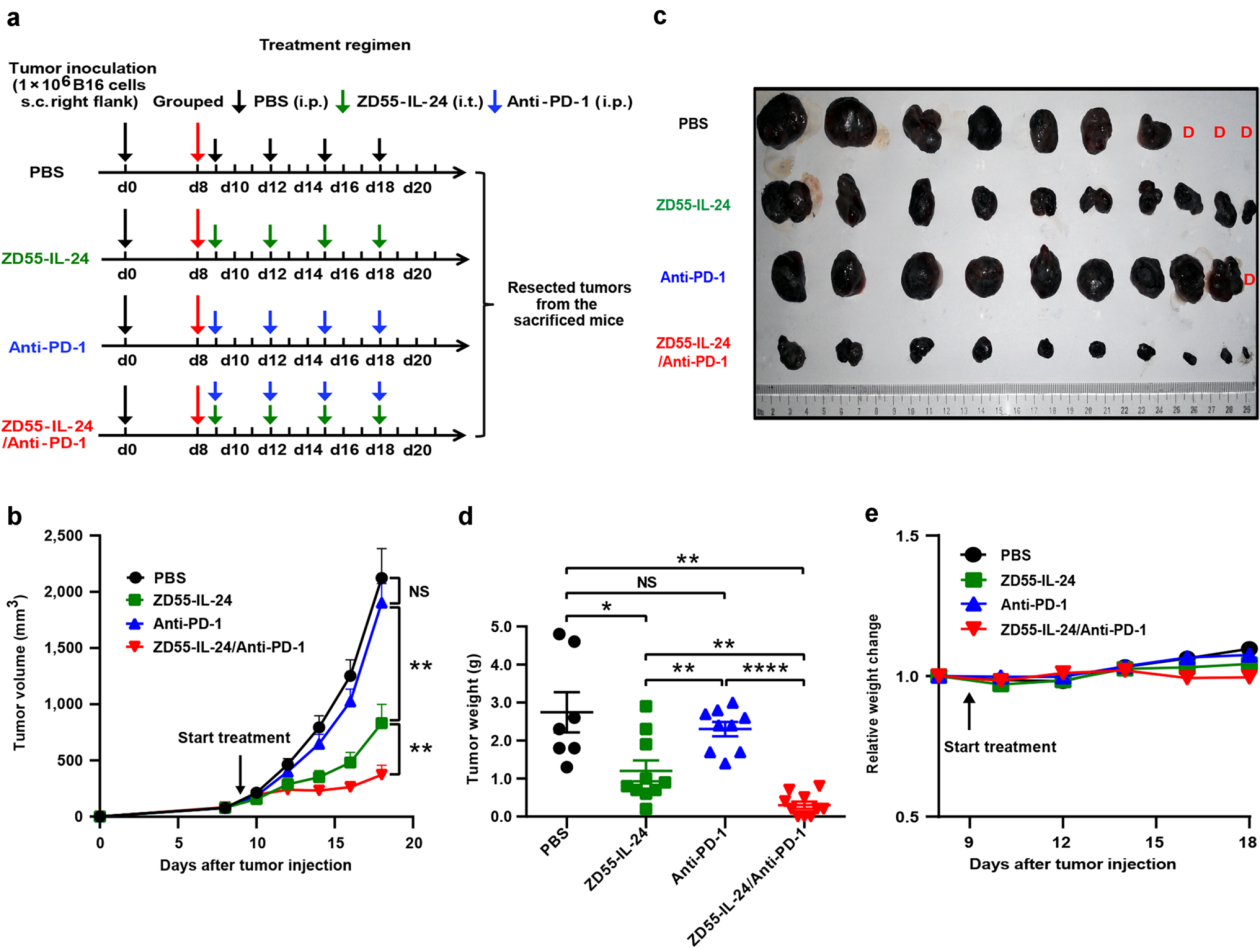
ZD55-IL-24 synergizes with PD-1 blockade to reject local established melanomas in B16-bearing immunocompetent mouse model. C57BL/6 mice were inoculated with 1 × 10^6^ B16 melanoma cells s.c. in the right flank and treated 9 d later (the average tumor volume was about 80 mm^3^) with PBS, anti-PD-1 antibody (0.2 mg/dose), ZD55-IL-24 (7.5 × 10^8^ PFU/dose), or the combination via intraperitoneal or intratumoral injection as indicated. **a** Scheme of tumor engraftment and treatments. **b** In vivo tumor growth curves. **c** Photograph of tumors resected from the sacrificed mice at the end of the experiment. **d** Weight of tumors resected from the sacrificed mice at the end of the experiment. **e** Body weight changes of mice monitored during the therapy period. *s.c.* subcutaneous injection, *i.t.* intratumoral injection, *i.p.* intraperitoneal injection, *D* Death, *n* = 10 C57BL/6 mice per group. Data are presented as Mean ± SEM

Female C57BL/6 mice 6 weeks of age were obtained from SLAC and quarantined for 2 weeks before tumor implantation. An inoculum of 1 × 10^6^ B16 cells was injected subcutaneously on the right flank of mice in 100 µL sterile PBS. When the subcutaneous tumor xenograft size reached about 80 mm^3^ (Day 8), the mice were randomized into four groups using R software, and treatments were initiated as indicated in Fig. 1a. ZD55-IL-24 was administered intratumorally at 50 μL per dose (7.5 × 10^8^ PFU/dose). Anti-PD-1 was administered intraperitoneally at 200 μg per dose in a volume of 100 μL antibody dilution buffer (BioXcell, IP0070). Tumor size (Volume = length × width^2^ × 0.5) and body weight were measured every 2 days, and mice were euthanized when the average tumor volume of PBS group exceeded 2,000 mm^3^. Photographs of the tumors resected from the sacrificed mice were taken immediately.

### Distant tumor therapy experiment

Female C57BL/6 mice 6 weeks of age were obtained from SLAC and quarantined for 2 weeks before tumor implantation. Tumors were implanted by injection of 1 × 10^6^ B16 cells in the right flank intradermally on day 0 and 2 × 10^5^ B16 cells in the left flank on day 4. When the average right flank tumor xenograft size reached about 80 mm^3^ (Day 11), the mice were randomized into 6 groups using R software, and treatments were initiated as indicated in Fig. 3a. ZD55-IL-24 was administered intratumorally at 50 μL per dose (7.5 × 10^8^ PFU/dose). Anti-PD-1 and isotype control antibody were administered intraperitoneally at 200 μg per dose in a volume of 100 μL antibody dilution buffer (BioXcell, IP0070). Tumor size (Volume = length × width^2^ × 0.5) as well as body weight were measured every 2 days, and mice were euthanized when the average right tumor volume of PBS group exceeded 2,000 mm^3^. Animal survival was also recorded every 2 days.

### Statistical analyses

Statistical analyses were performed using GraphPad Prism 6.0. Comparisons between two groups were performed using Student’s t-test. Comparison of multiple groups was performed by analysis of variance (ANOVA). Survival curves were analyzed using log-rank (Mantel-Cox) test. Differences were considered significant at *P* < 0.05 (*P < 0.05, **P < 0.01, ***P < 0.001, ****P < 0.0001, NS, not significant).

## Results

### Synergistic effect of ZD55-IL-24 and PD-1 blockade in primary tumors

To examine the feasibility and efficacy of ZD55-IL-24 therapy combined with anti-PD-1 therapy, we compared monotherapy with ZD55-IL-24 alone, or anti-PD-1 alone versus dual therapy with ZD55-IL-24 plus anti-PD-1 in B16-bearing immunocompetent mouse model. B16 melanoma cells (10^6^) were injected subcutaneously (s.c.) to the right flank of C57BL/6 mice and allowed to grow for 8 days before initiation of therapy, resulting in tumor sizes of ~ 80 mm^3^ at the initial time of treatment. ZD55-IL-24 was administered intratumorally (i.t.), and anti-PD-1 antibody was injected intraperitoneally (i.p.). Therapies were given every 3 days, with both ZD55-IL-24 and anti-PD-1 antibody being administered four times (Fig. 1a).

Although the therapeutic effects were observed in both monotherapies, especially the robust therapeutic effect of ZD55-IL-24 therapy alone, the combined therapy further slowed the growth of established B16 melanomas compared with either therapy alone (Fig. 1b). The tumor volume and weight in the combination therapy group were 371.5379 ± 84.83121 mm^3^ and 0.3 ± 0.09189 g, whereas the PBS-treated group was 2122.229 ± 261.3108 mm^3^ and 2.743 ± 0.5291 g, the ZD55-IL-24-treated group was 830.6364 ± 167.3815 mm^3^ and 1.2 ± 0.2737 g, and the anti-PD-1-treated group was 1903.805 ± 169.4441 mm^3^ and 2.3 ± 0.1878 g. The tumors of the combination treatment group were significantly smaller than the tumors in the other groups (Fig. 1c, d), indicating that the synergistic potential of ZD55-IL-24 and PD-1 blockade. Of note, despite high anti-melanoma efficacy, the combination of ZD55-IL-24 and PD-1 blockade was associated with no significant systemic toxicity, as mice did not show significant weight or hair loss (Fig. 1e).

### ZD55-IL-24 can improve the low tumor immune infiltration in primary tumors of the anti-PD-1-treated mice

**Fig. 2:**
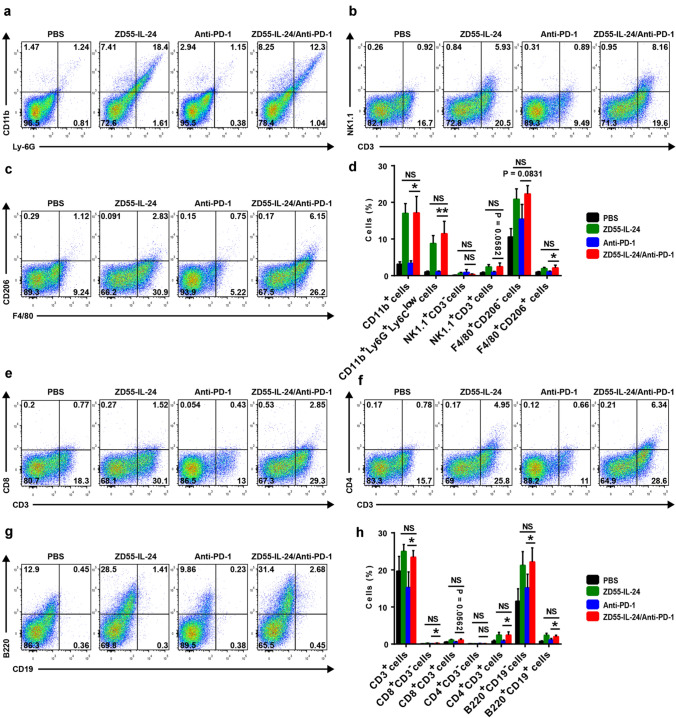
ZD55-IL-24 helps PD-1 blockade to overcome the limitation of relatively low tumor immune infiltration in local established tumors. C57BL/6 mice were treated as indicated in Fig. 1a, and then the tumor cells were isolated for flow cytometry analysis. Tumor immune infiltration of innate immune cells in local tumors. **a** Representative flow cytometry plots of tumor-infiltrating total myeloid cells (CD11b^+^) and neutrophils (CD11b^+^Ly-6G^+^Ly-6C^low^). **b** Representative flow cytometry plots of tumor-infiltrating NK cells (NK1.1^+^CD3^−^) and NKT cells (NK1.1^+^CD3^+^). **c** Representative flow cytometry plots of tumor-infiltrating M1 macrophages (F4/80^+^CD206^−^) and M2 macrophages (F4/80^+^CD206^+^). **d** Percentages of various innate immune cells in local tumors. Tumor immune infiltration of adaptive immune cells in local tumors. **e** Representative flow cytometry plots of tumor-infiltrating total *T* cells (CD3^+^), CD8^+^CD3^−^ cells and CD8^+^ *T* cells (CD8^+^CD3^+^). **f** Representative flow cytometry plots of tumor-infiltrating CD4^+^CD3^−^ cells and CD4^+^ *T* cells (CD4^+^CD3^+^). **g** Representative flow cytometry plots of tumor-infiltrating plasma cells (B220^+^CD19^−^) and B cells (B220^+^CD19^+^). **h** Percentages of various adaptive immune cells in local tumors. Mean ± SEM is shown. Data represent cumulative results from seven independent experiments

To understand the mechanism of improved anti-melanoma efficacy with the combination therapy, we analyzed cellular infiltrates in treated tumors using flow cytometry. Compared with the PBS control group, the anti-PD-1 therapy only group had no significant effect on the promotion of tumor immune infiltration in local tumors (Fig. 2d, h). In contrast, a substantial increase of tumor immune infiltration was observed in the ZD55-IL-24 monotherapy group. The combination therapy showed an enhanced tumor immune infiltration compared with anti-PD-1 therapy alone, albeit no further enhancement compared with ZD55-IL-24 therapy alone (Fig. 2d, h). The enhanced immune infiltrates in primary tumors were characterized by increase in innate immune compartment, including overall myeloid cells, neutrophils, natural killer T (NKT) cells, M1 macrophages and M2 macrophages (Fig. 2a–d), and the adaptive compartment, including overall *T* cells, CD8^+^CD3^−^ cells, CD8^+^ T cells, CD4^+^ *T* cells, plasma cells and B cells (Fig. 2e–h). Among the above immune cells, the antitumor effect of neutrophils, NKT cells, M1 macrophages, CD8^+^ *T* cells and CD4^+^ *T* cells has been well-documented [5, 23–27]. These results indicated that ZD55-IL-24 was able to help PD-1 blockade to overcome the weakness of relatively low tumor immune infiltration in primary tumors.

Given the negative role of regulatory *T* (*T*_reg_) cells in antitumor immunity [28, 29], we next further analyzed the infiltration of *T*_reg_ cells in local established tumors and calculated the conventional CD4^+^FoxP3^−^ *T* (*T*_conv_) cells to *T*_reg_ cells ratio, which has been previously demonstrated to be a marker of a favorable immunological response to immunotherapy [23, 30]. Both ZD55-IL-24 only and PD-1 blockade only caused a significant enhancement of *T*_reg_ cells in local tumors compared with the PBS control group, and the B16 tumors treated with combination therapy showed a further higher accumulation of *T*_reg_ cells than those tumors treated by PD-1 blockade alone, albeit no further higher accumulation than ZD55-IL-24 therapy alone (Supplementary Figure 1a, b). Although there is a significant increase in the percentages of *T*_reg_ cells which suppressed tumor-specific immune responses in ZD55-IL-24 therapy alone, anti-PD-1 therapy alone and the combination therapy groups, there is also a substantial increase in the percentages of *T*_conv_ cells, with marked enhancement of the CD4 effector to *T*_reg_ ratio (Supplementary Figure 1a, c), suggesting that both ZD55-IL-24 treatment and anti-PD-1 treatment can elicit substantial remodeling of the immunosuppressive tumor microenvironment with contributions to the immunotherapy. However, the *T*_conv_/*T*_reg_ ratio of the combination treatment group did not show statistically significant differences compared to ZD55-IL-24 therapy alone or anti-PD-1 therapy alone groups (Supplementary Figure 1a, c), suggesting that ZD55-IL-24 was unable to help PD-1 blockade to further improve the tumor immune microenvironment.

### Synergistic effect of ZD55-IL-24 and PD-1 blockade in distant tumors

**Fig. 3:**
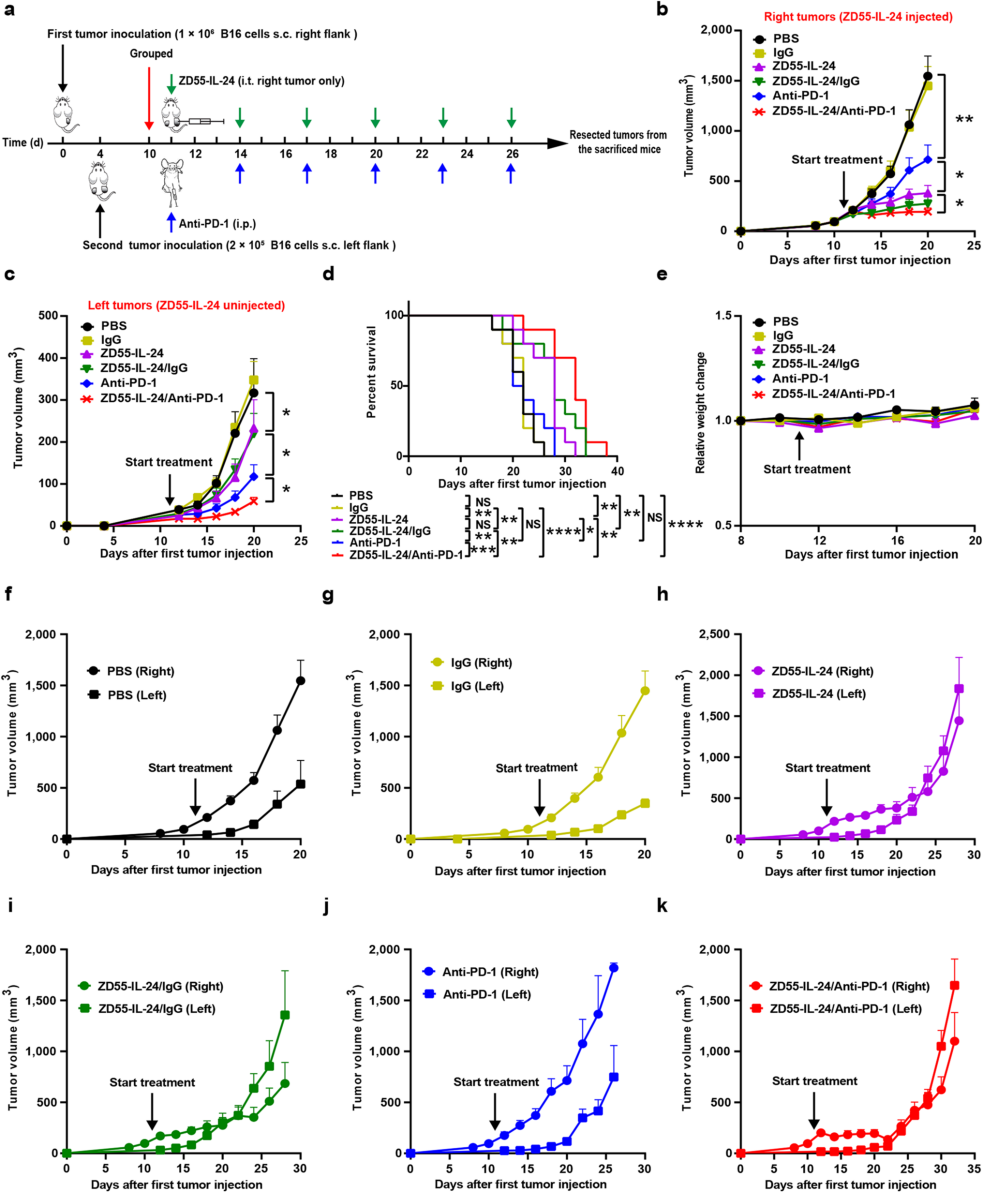
ZD55-IL-24 also synergizes with PD-1 blockade to reject distant uninjected melanomas in B16-bearing immunocompetent mouse model. C57BL/6 mice were inoculated with 1 × 10^6^ B16 cells s.c. in the right flank on day 0 and 2 × 10^5^ B16 cells s.c. in the left flank on day 4. The B16-bearing C57BL/6 mice were subsequently treated with PBS, isotype IgG (0.2 mg/dose), anti-PD-1 antibody (0.2 mg/dose), ZD55-IL-24 (7.5 × 10^8^ PFU/dose), or the combination via intraperitoneal or intratumoral injection as indicated, starting when the average tumor volume was about 80 mm^3^. **a** Scheme of tumor engraftment and treatments. **b** Growth of local ZD55-IL-24-injected (right flank) and **c** distant ZD55-IL-24-uninjected (left flank) tumors. **d** Overall survival. **e** Body weight changes of the mice monitored during the therapy period. **f** Local and distant tumor growth curves of Group PBS, **g** Group IgG, **h** Group ZD55-IL-24, **i** Group ZD55-IL-24/IgG, **j** Group anti-PD-1 and **k** Group ZD55-IL-24/anti-PD-1, respectively. *s.c.* subcutaneous injection, *i.t.* intratumoral injection, *i.p.* intraperitoneal injection, *n* = 10 C57BL/6 mice per group. Data are presented as Mean ± SEM

Motivated by the synergy observed between ZD55-IL-24 and PD-1 blockade in local tumors, we further sought to evaluate whether ZD55-IL-24 was also able to synergize with PD-1 blockade to reject the distant uninjected tumors in B16-bearing immunocompetent mouse model using a bilateral flank B16 tumor model [23]. We inoculated 1 × 10^6^ B16 melanoma cells into the right flank of C57BL/6 mice intradermally on day 0, and four days later the mice were challenged with a second injection of 2 × 10^5^ B16 melanoma cells in the left flank (Fig. 3a). Six fractionated doses of ZD55-IL-24 (7.5 × 10^8^ PFU/dose) and anti-PD-1 (0.2 mg/dose) were administered intratumorally or intraperitoneally every other two days. ZD55-IL-24 was selectively applied only to the right flank tumor lesions (local tumor), while a contralateral tumor was set without ZD55-IL-24 injection (distant tumor).

In the bilateral flank B16 tumor model, ZD55-IL-24 treatment in combination with PD-1 blockade significantly slowed not only local (Fig. 3b) but also distant (Fig. 3c) tumor growth compared to untreated or animals receiving isotype IgG, ZD55-IL-24 or anti-PD-1 monotherapy, or combination therapy with ZD55-IL-24 and isotype IgG. Consistent with the tumor growth data, the survival of mice receiving ZD55-IL-24 plus anti-PD-1 therapy was significantly prolonged compared with other treatments, although the ZD55-IL-24 monotherapy and ZD55-IL-24 plus isotype IgG therapy were also able to greatly prolong the shortened lifespan of mice (Fig. 3d). In addition, we did not observe any obvious toxicity such as weight or hair loss in mice receiving combination treatment in the bilateral flank B16 tumor model as well (Fig. 3e). These data collectively showed that the combined therapy with ZD55-IL-24 and PD-1 blockade improved antitumor responses against not only local but also distant B16 tumors in B16-bearing immunocompetent mouse model. Intriguingly, we noticed that the distant tumors grew considerably faster than local tumors in the ZD55-IL-24, ZD55-IL-24/IgG and ZD55-IL-24/anti-PD-1 groups, whereas the growth of local and distant tumors was practically identical in the PBS, IgG and anti-PD-1 groups (Fig. 3f–k), indicating that ZD55-IL-24 appeared much more effective in local tumors than distant tumors, whereas anti-PD-1 appeared identical effective in both local and distant tumors, which was consistent with the fact that anti-PD-1 antibody was administered intraperitoneally.

### ZD55-IL-24 can also improve the low immune cell infiltrates in distant tumors of the anti-PD-1-treated mice

**Fig. 4:**
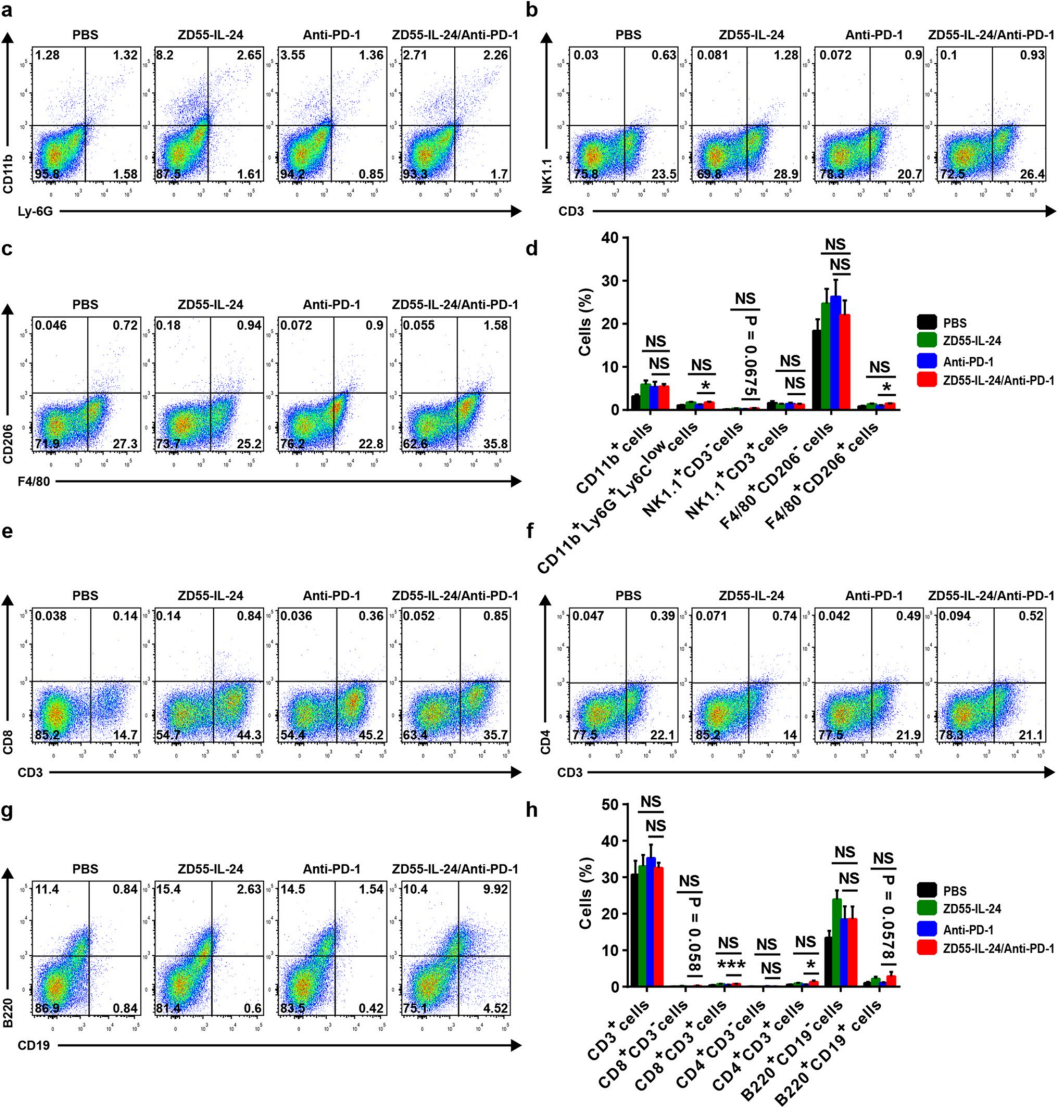
ZD55-IL-24 helps PD-1 blockade to overcome the weakness of relatively low immune cell infiltration in not only local injected but also distant uninjected tumors. C57BL/6 mice were treated as indicated in Fig. 3a, and then the left flank tumors were resected from the sacrificed mice for flow cytometry analysis. Tumor immune infiltration of innate immune cells in distant tumors. **a** Representative flow cytometry plots of tumor-infiltrating total myeloid cells and neutrophils in distant tumors. **b** Representative flow cytometry plots of tumor-infiltrating NK cells and NKT cells in distant tumors. **c** Representative flow cytometry plots of tumor-infiltrating M1 macrophages and M2 macrophages in distant tumors. **d** Percentages of various innate immune cells in distant tumors. Tumor immune infiltration of adaptive immune cells in distant tumors. **e** Representative flow cytometry plots of tumor-infiltrating total T cells, CD8^+^CD3^−^ cells and CD8^+^ *T* cells in distant tumors. **f** Representative flow cytometry plots of tumor-infiltrating CD4^+^CD3^−^ cells and CD4^+^ *T* cells in distant tumors. **g** Representative flow cytometry plots of tumor-infiltrating plasma cells and B cells in distant tumors. **h** Percentages of various adaptive immune cells in distant tumors. Mean ± SEM is shown. Data represent cumulative results from seven independent experiments

Based on the synergistic anti-melanoma effect in distant tumors, we thus reasoned that ZD55-IL-24 also helped PD-1 blockade to overcome the weakness of relatively low immune cell infiltration in distant tumors. To further examine the infiltrating of immune cells in distant tumors, we next collected and processed distal tumors for flow cytometry analysis. Our flow cytometric results revealed that the immune cell infiltrates in distant tumors were similar to local tumors (Fig. 4d, h). A great increase in the innate immune compartment, which included total myeloid cells, neutrophils, natural killer (NK) cells, M1 macrophages and M2 macrophages (Fig. 4a–d), and the adaptive immune compartment, which included CD8^+^ T cells, CD4^+^ *T* cells, plasma cells and B cells (Fig. 4e–h), was observed in distant tumors of the mice treated with ZD55-IL-24 monotherapy. However, only a modest increase in both innate and adaptive immune compartment was observed in distant tumors of the mice treated with anti-PD-1 monotherapy. As expected, the combination therapy further enhanced tumor immune infiltration in distant tumors compared with anti-PD-1 therapy alone, despite no statistically significant difference was observed when compared with ZD55-IL-24 therapy alone (Fig. 4d, h), which was in line with the observation in local tumors. Compared with the anti-PD-1 therapy group, a great increase in neutrophils, NK cells, M2 macrophages, CD8^+^ CD3^−^ cells, CD8^+^ T cells, CD4^+^ T cells and B cells was observed in distant tumors of the mice treated with combination therapy (Fig. 4a–h). These results suggested that ZD55-IL-24 was able to help PD-1 blockade to overcome the weakness of relatively low tumor immune infiltration in not only local but also distal tumors in B16-bearing immunocompetent mouse model.

In addition, we also analyzed the infiltration of *T*_reg_ cells in distant tumors and calculated the *T*_conv_/*T*_reg_ ratio. Unlike in local tumors, there was no significant change in *T*_reg_ cells in distant tumors among the different treatment groups (Supplementary Figure 2a, b). The *T*_conv_/*T*_reg_ ratio, however, was significantly increased in distant tumors of the ZD55-IL-24 therapy alone, anti-PD-1 therapy alone and combination therapy groups (Supplementary Figure 2a, c), suggesting that both ZD55-IL-24 treatment and anti-PD-1 treatment can also elicit remodeling of the immunosuppressive tumor microenvironment in distant tumors with contributions to the immunotherapy of distant tumors. In agreement with what we observed in local tumors (Supplementary Figure 1a, c), the combination therapy also failed to further increase the *T*_conv_/*T*_reg_ ratio in distant tumors compared to ZD55-IL-24 therapy alone or anti-PD-1 therapy alone (Supplementary Figure 2a, c), suggesting that ZD55-IL-24 was unable to help PD-1 blockade to further improve the tumor immune microenvironment in distant tumors as well.

### ZD55-IL-24 cannot help PD-1 blockade to further facilitate immune cell recruitment and activation in spleens

**Fig. 5:**
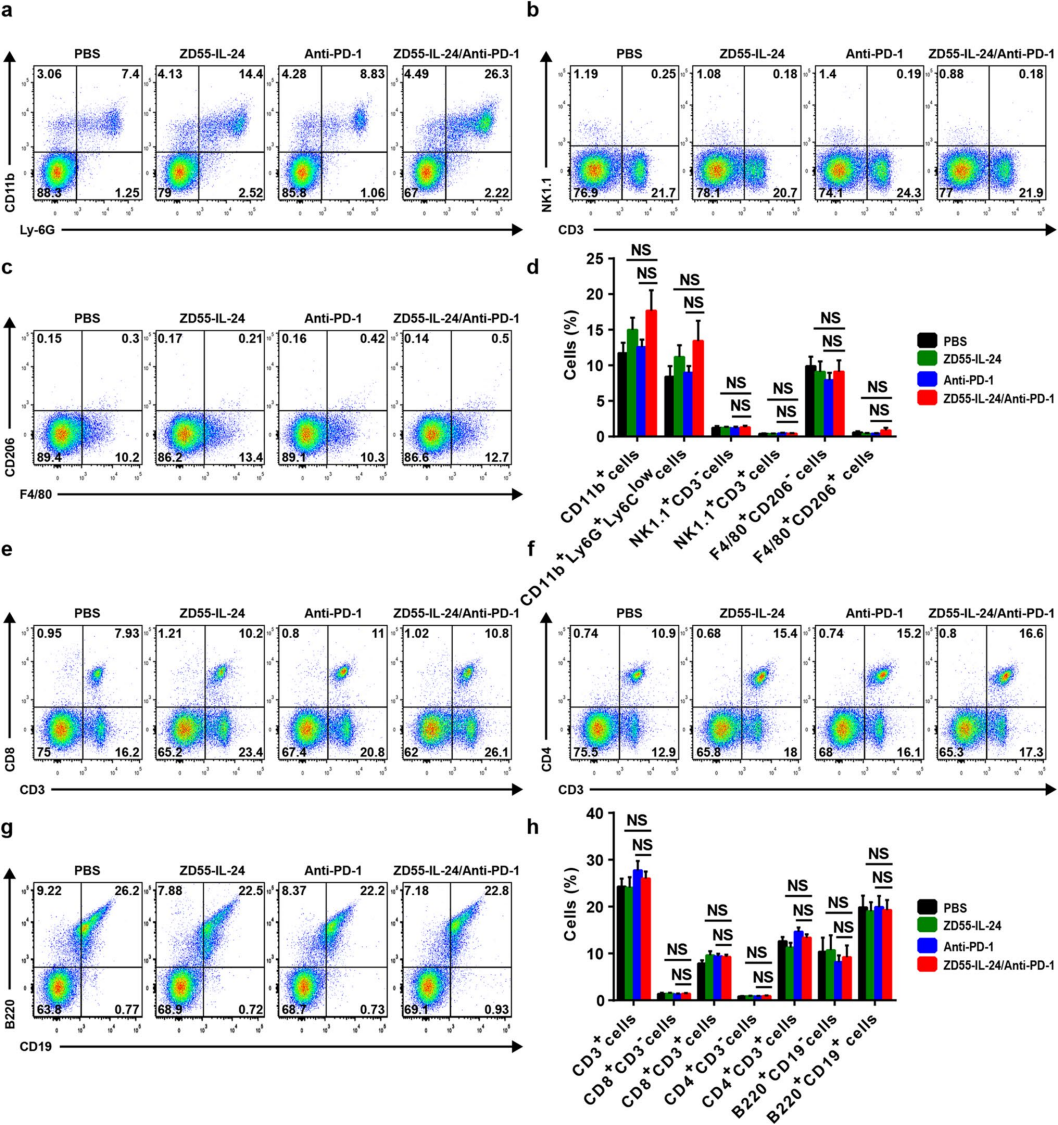
ZD55-IL-24 fails to help PD-1 blockade to further facilitate immune cell recruitment and activation in spleens. C57BL/6 mice were treated as indicated in Fig. 3a, and then the spleen cells were isolated for flow cytometry analysis. Recruitment and activation of innate immune cells in spleens. **a** Representative flow cytometry plots of tumor-infiltrating total myeloid cells and neutrophils in spleens. **b** Representative flow cytometry plots of tumor-infiltrating NK cells and NKT cells in spleens. **c** Representative flow cytometry plots of tumor-infiltrating M1 macrophages and M2 macrophages in spleens. **d** Percentages of various innate immune cells in spleens. Recruitment and activation of adaptive immune cells in spleens. **e** Representative flow cytometry plots of tumor-infiltrating total T cells, CD8^+^CD3^−^ cells and CD8^+^ *T* cells in spleens. **f** Representative flow cytometry plots of tumor-infiltrating CD4^+^CD3^−^ cells and CD4^+^ *T* cells in spleens. **g** Representative flow cytometry plots of tumor-infiltrating plasma cells and B cells in spleens. **h** Percentages of various adaptive immune cells in spleens. Mean ± SEM is shown. Data represent cumulative results from seven independent experiments

The spleen, where mature naive lymphocytes are maintained, collects antigen from the blood and initiates the systemic immune responses [31]. Motivated by the immune infiltration-promoting signature in distant tumors (Fig. 4a–h), we proceeded to investigate whether ZD55-IL-24 was able to further increase immune cell recruitment and activation in spleens of the anti-PD-1-treated mice. In spleens, other than an increased percentage of CD8^+^ T cells with ZD55-IL-24 or anti-PD-1-treated mice versus PBS control, there was no significant change among the different treatment groups (Fig. 5a–h), indicating that ZD55-IL-24 was unable to help PD-1 blockade to further facilitate immune cell recruitment and activation in the spleen. Likewise, we also analyzed the recruitment and activation of *T*_reg_ cells in spleens and calculated the *T*_conv_/*T*_reg_ ratio. However, no significant difference in both *T*_reg_ cells and *T*_conv_/*T*_reg_ ratio was observed between the treatment groups (Supplementary Figure 3a–c), showing that ZD55-IL-24 was unable to help PD-1 blockade to reverse the immunosuppressive milieu in the spleen.

### ZD55-IL-24 can help PD-1 blockade to overcome the weakness of relatively low tumor immune recognition

**Fig. 6:**
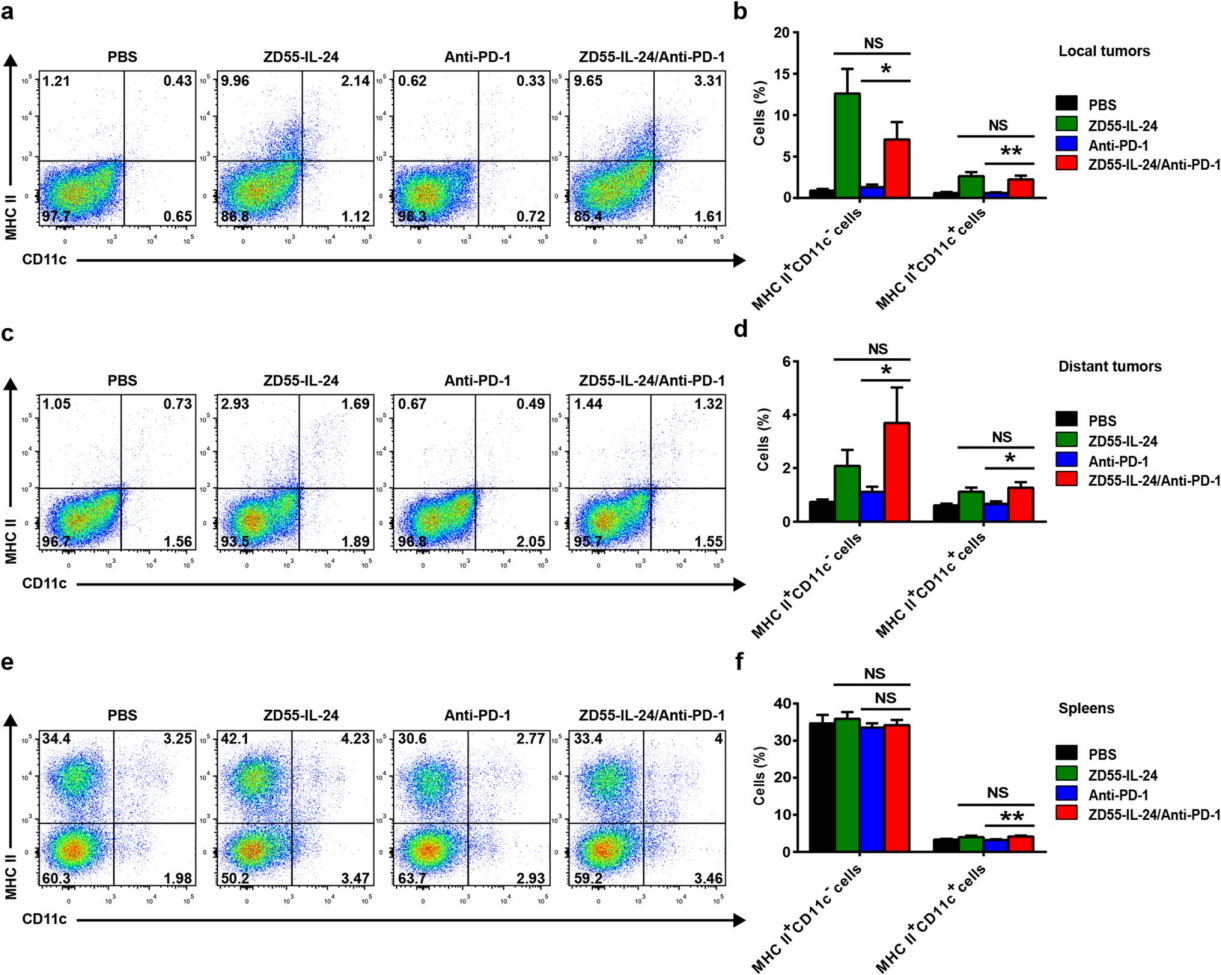
ZD55-IL-24 helps PD-1 blockade to overcome the limitation of relatively low tumor immune recognition in B16-bearing immunocompetent mouse model. C57BL/6 mice were treated as indicated in Fig. 3a, and then the tumor and spleen cells were isolated for flow cytometry analysis. **a** Representative flow cytometry plots of tumor-infiltrating MHC II^+^CD11c^−^ APCs and DCs (MHC II^+^CD11c^+^) in local tumors. **b** Percentages of MHC II^+^CD11c^−^ APCs and DCs in local tumors. **c** Representative flow cytometry plots of tumor-infiltrating MHC II^+^CD11c^−^ APCs and DCs in distant tumors. **d** Percentages of MHC II^+^CD11c^−^ APCs and DCs in distant tumors. **e** Representative flow cytometry plots of MHC II^+^CD11c^−^ APCs and DCs in spleens. **f** Percentages of MHC II^+^CD11c^−^ APCs and DCs in spleens. Mean ± SEM is shown. Data represent cumulative results from seven independent experiments

Immune responses are initiated when antigen-presenting cells (APCs), especially dendritic cells (DCs), recognize antigens [31]. However, tumor cells tend to be self-origin and are inherently not very immunogenic, hence failure to be recognized by APCs is generally the major obstacle for cancer immunotherapy including PD-1 blockade therapy [27, 31]. Thus, we finally explored the impact of combination therapy on the tumor immune recognition. Both MHC II^+^CD11c^−^ APCs and DCs were significantly increased in local (Fig. 6a, b) and distant (Fig. 6c, d) tumors in ZD55-IL-24-treated mice, but not in anti-PD-1-treated mice, indicating that ZD55-IL-24 was able to improve tumor immune recognition in both local and distant tumors, while PD-1 blockade failed to achieve this effect. Similarly, ZD55-IL-24 treatment alone led to a small but statistically significant increase of DCs in spleens as well, despite there was no difference in MHC II^+^CD11c^−^ APCs (Fig. 6e, f), further indicating that ZD55-IL-24 but not PD-1 blockade was able to improve tumor immune recognition in spleens. With the exception of MHC II^+^CD11c^−^ APCs in spleens, a substantial increase of MHC II^+^CD11c^−^ APCs and DCs in local and distant tumors as well as spleens was observed in the combination therapy group when compared to anti-PD-1 monotherapy group, albeit no further increase compared with ZD55-IL-24 monotherapy group (Fig. 6b, d, f). Taken together, these data indicated that ZD55-IL-24 rather than PD-1 blockade was able to improve tumor immune recognition in both local and distant tumors as well as spleens, and ZD55-IL-24 could help PD-1 blockade to overcome the limitation of relatively low tumor immune recognition, contributing to the observed synergistic effect in B16-bearing immunocompetent mouse model.

## Discussion

There is a growing body of evidence to support combinatorial approaches that merge immune checkpoint blockade therapy with oncolytic virotherapy [32–36]. ZD55-IL-24 is an armed oncolytic adenovirus type 5 analogous but superior to the famous ONYX-015 and H101 (Oncorine). ONYX-015 has been evaluated in several clinical trials, though limited responses were reported and clinical development was halted in 2003 [14]. H101 has been approved for clinical use for head and neck cancer in China in 2005 [14]. Here, we demonstrate for the first time that our ZD55-IL-24 has synergistic activity with anti-PD-1 therapy in B16-bearing immunocompetent mouse model. These data support further evaluation of ZD55-IL-24 as a combination partner in clinical trials.

**Fig. 7:**
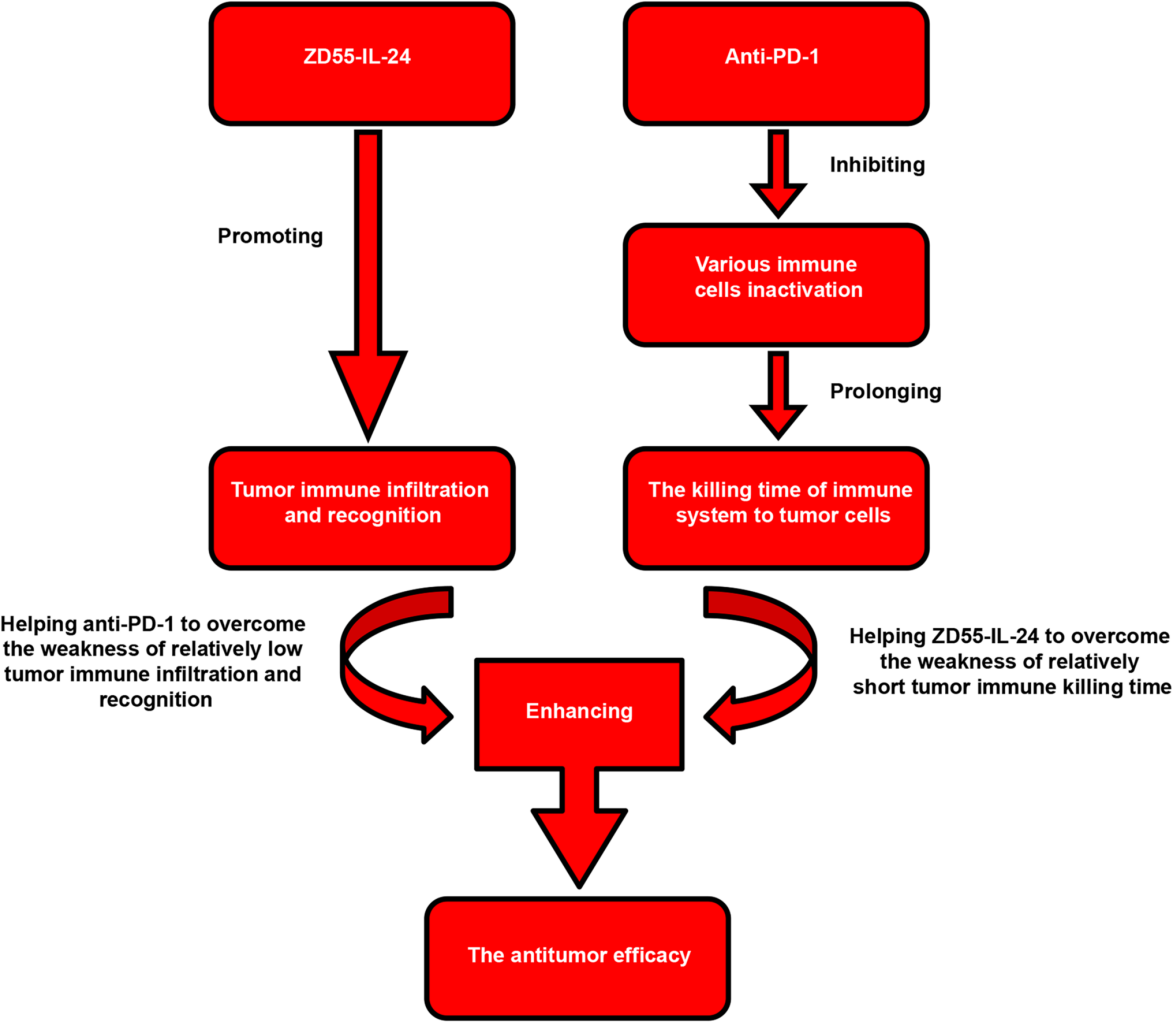
Mechanistic model of combination therapy with ZD55-IL-24 and PD-1 blockade

In this study, we found that anti-PD-1 treatment in fact had no apparent effect on tumor immune infiltration and recognition. ZD55-IL-24 treatment, however, was able to greatly promote tumor immune infiltration and recognition at the injected tumor site and in regression of distant tumors as well as in spleens. These results indicated that ZD55-IL-24 can help PD-1 blockade to overcome the limitation of relatively low tumor immune infiltration and recognition. On the other hand, PD-1 blockade is primarily believed to inhibit various effector immune cells inactivation in the effector phase within tissue and tumors, and thus prolonging the killing time of immune system to tumor cells [37]. However, ZD55-IL-24 fails to do this. Therefore, PD-1 blockade can help ZD55-IL-24 to overcome the limitation of relatively short tumor killing time. In this way, ZD55-IL-24 and PD-1 blockade achieve their synergistic therapeutic effect (Fig. 7).

The synergistic antitumor effect between oncolytic viruses and PD-1 blockade in B16-bearing immunocompetent mouse model has been well-documented [38–42]. Consistent with these reports, our results indeed demonstrate that ZD55-IL-24 synergizes with PD-1 blockade to reject not only local injected tumors but also distant uninjected tumors in B16-bearing immunocompetent mouse model. It is generally thought that the oncolytic virus infection can result in immunogenic tumor cell death and release the essential elements such as tumor-associated antigens, viral pathogen-associated molecular patterns, cellular danger-associated molecular pattern signals and cytokines such as type I interferons, which result in the generation of antitumor immunity [14, 23, 32, 43]. Thus, the induction of antitumor immunity by oncolytic viruses was thought to depend on successful viral infection, induction of immunogenic tumor cell death, and release of the essential elements. However, our recent research confirmed that ZD55-IL-24 can induce antitumor immunity in B16-bearing immunocompetent mouse model in fact not due to its ability to lyse tumor cells and release the essential elements, but due to its ability to turn the tumor cells from the unrecognizable “self” state into the recognizable “nonself” state without tumor cell death [44]. Hence, our previous and current results together indicate that ZD55-IL-24 can turn the tumor cells from the “self” state into the “nonself” state without tumor cell death, thus make the tumor cells easy to be recognized by original host immune system, finally help PD-1 blockade to reject melanomas in B16-bearing immunocompetent mouse model. The synergetic mechanism between ZD55-IL-24 and PD-1 blockade is somewhat different from the synergetic mechanism which we previously thought. Notably, we here find that synergy is also observed even though the oncolytic virus is unable to successfully infect and directly lyse tumor cells, suggesting that the synergistic effect between oncolytic viruses and PD-1 blockade probably depends on the capability of oncolytic viruses to turn the tumor cells from the “self” state into the “nonself” state, rather than the capability of oncolytic viruses to lyse tumor cells.

Our study does present several limitations. First of all, immunologic assessments of oncolytic adenoviruses are known to be challenging as good model systems that allow for both viral replication and immunologic analyses are limited. Indeed, our results indicated that ZD55-IL-24 is unable to successfully infect and express exogenous IL-24 gene in B16 cells, the IL-24 gene harbored in ZD55-IL-24 viral genome in fact plays no role in B16-bearing immunocompetent mouse model [44]. Therefore, the observed synergistic therapeutic effect between ZD55-IL-24 and PD-1 blockade here is practically caused only by the viral vector. However, IL-24 also displays direct and indirect antitumor activity through induction of autophagy and cancer-specific apoptosis, stimulation of an antitumor immune response, inhibition of angiogenesis, and sensitization of cancer cells to radiation-, chemotherapy- and antibody-induced killing [45]. Thus, the synergistic antitumor effect between IL-24 and PD-1 blockade is unable to be observed in current study. However, it is reasonable to surmise that a more robust synergistic effect between ZD55-IL-24 and PD-1 blockade will be observed when the IL-24 gene works. Second, it is well known that most murine tissues are not supportive of human adenovirus replication. Data obtained from mouse model are perhaps different from patient situation. These challenges, however, can be addressed in clinical trials. Hence, further investigation in patients may yield important information.

In our study, we observed that the anti-melanoma efficacy of ZD55-IL-24 in local tumors is far higher than that of anti-PD-1 antibody in B16-bearing immunocompetent mouse model (Figs. 1a–e and 3b), indicating ZD55-IL-24 has an obvious advantage in antitumor efficacy. Besides the much higher antitumor efficacy, another advantage of ZD55-IL-24 is the far lower production cost compared with anti-PD-1 antibody (the cost of ZD55-IL-24 in this study is less than $ 1, but the cost of anti-PD-1 antibody is more than $ 1000). The manufacturing process of ZD55-IL-24, which is based on viral replication and purification, is much simpler; in contrast, anti-PD-1 antibody has to be produced via much more complex process, which is based on protein expression and purification. Less expensive production cost may help to reduce drug price. In addition, the dramatic response of anti-PD-1 therapy is often restricted to a minority of patients [3, 5, 37], yet ZD55-IL-24 therapy appears less individual variance (data not shown). Nevertheless, ZD55-IL-24 does present some limitations. Since it must be administered intratumorally at present, ZD55-IL-24 is only suitable for treating those few tumors with visible lesions such as melanoma and in fact difficult to be used to treat the vast majority of tumors in clinic. Albeit with far lower efficacy and response rate as well as much higher cost, anti-PD-1 antibody can be administered systemically and thus can be theoretically used to treat almost all tumors, which is an incomparable advantage. Moreover, ZD55-IL-24 still presents some limitations even for melanoma treatment. For example, the dramatic anti-melanoma efficacy of ZD55-IL-24 is merely confined to local tumors, but substantially reduced in distant tumors and extremely lower than anti-PD-1 antibody (Fig. 3b, c, f–k). Unlike ZD55-IL-24, the anti-melanoma efficacy of anti-PD-1 antibody is identical in both local and distant tumors. ZD55-IL-24 and PD-1 blockade can help each other overcome their limitations. Therefore, although ZD55-IL-24 alone or PD-1 blockade alone might be successful in melanoma therapy, combination therapy might be much more reliable in future clinical therapy.

### Supplementary information

Below is the link to the electronic supplementary material.

Supplementary file1 (PDF 1001 kb)

## Data Availability

Correspondence and requests for data and materials should be addressed to X.-Y.L., H.-J.H., or A.-M.N. ZD55-IL-24 was supplied by Yuansong Biotech Inc. under a material transfer agreement with Shanghai Institutes for Biological Sciences, Chinese Academy of Sciences, China.
