# Supplementary material for: Enhanced anti-melanoma efficacy through a combination of the armed oncolytic adenovirus ZD55-IL-24 and immune checkpoint blockade in B16-bearing immunocompetent mouse model
Source: Cancer Immunol Immunother. 2021 Apr 26;70(12):3541–55. doi: 10.1007/s00262-021-02946-z (PMC8571158; doi:10.1007/s00262-021-02946-z)
Supplement: Supplementary file 1 — Supplementary file1 (PDF 1001 kb) [file 262_2021_2946_MOESM1_ESM.pdf]

Supplementary Figures

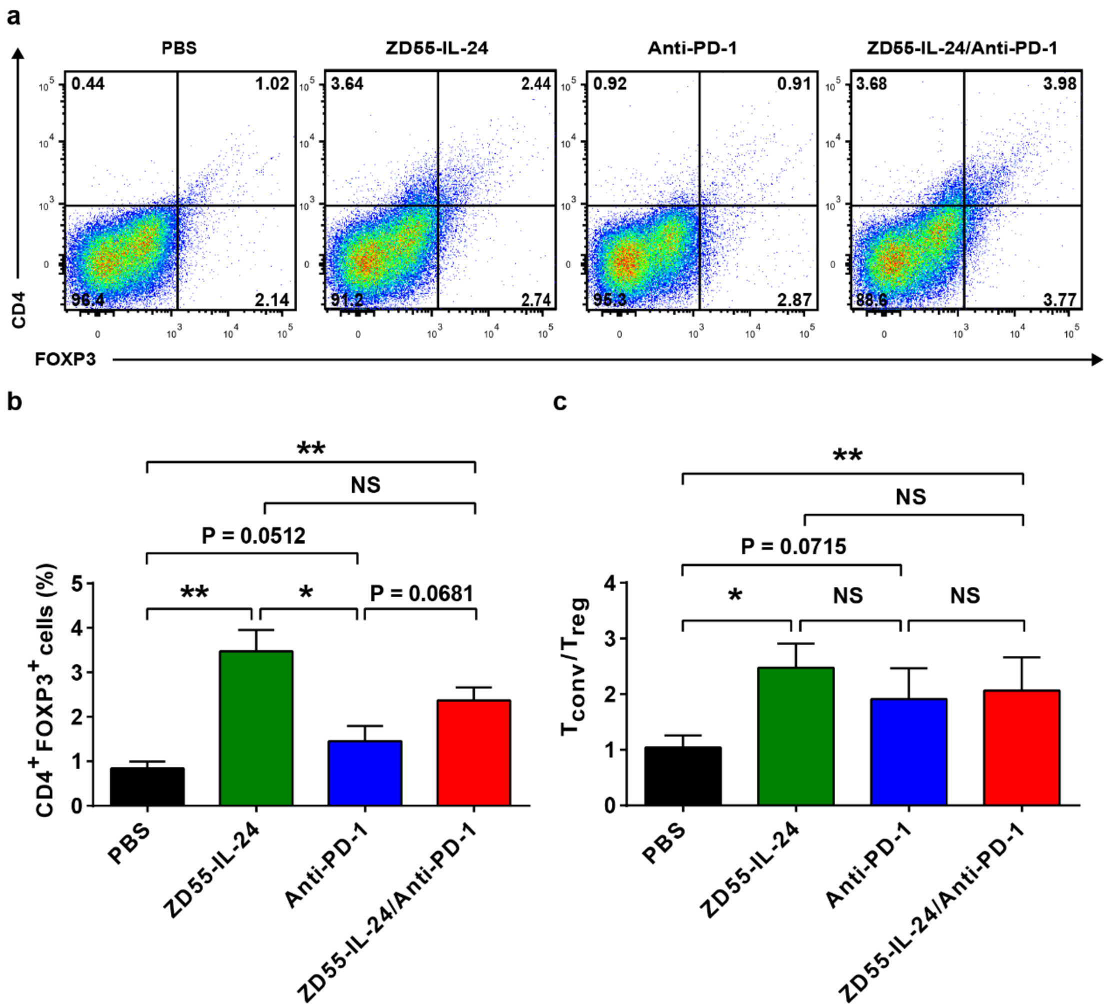

Supplementary Figure 1 ZD55-IL-24 is unable to help PD-1 blockade to further increase the T_{conv}/T_{reg} ratio in local tumors. C57BL/6 mice were treated as indicated in **Fig. 1a**, and then the tumor cells were isolated for flow cytometry analysis. **(a)** Representative flow cytometry plots of tumor-infiltrating T_{conv} cells ($CD4^{+}FOXP3^{-}$) and T_{reg} cells ($CD4^{+}FOXP3^{+}$). **(b)** Percentages of T_{reg} cells in tumors. **(c)** Calculated T_{conv}/T_{reg} ratio. Mean ± SEM is shown. Data represent cumulative results from seven independent experiments

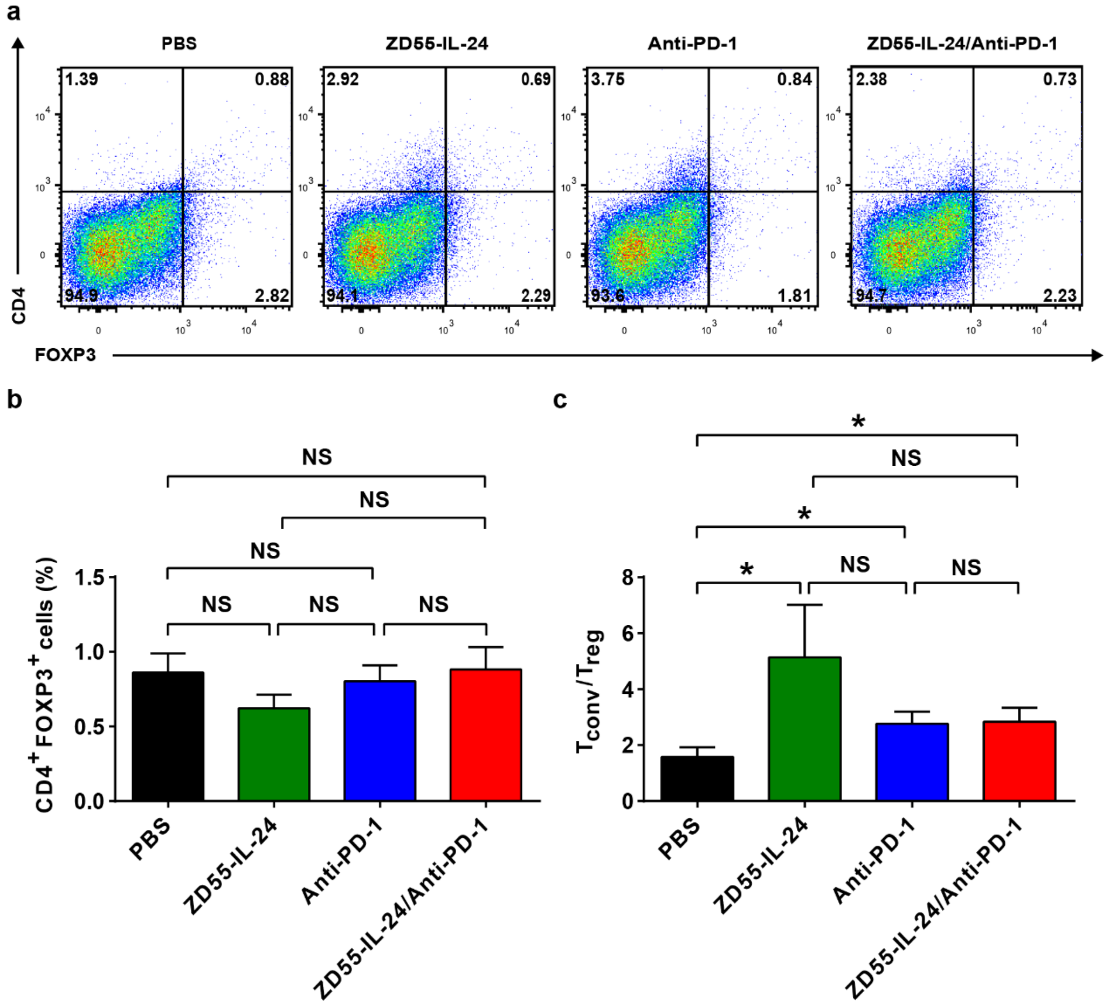

Supplementary Figure 2 ZD55-IL-24 is also unable to help PD-1 blockade to further increase the T_{conv}/T_{reg} ratio in distant tumors. C57BL/6 mice were treated as indicated in **Fig. 3a**, and then the left flank tumors were resected from the sacrificed mice for flow cytometry analysis. **(a)** Representative flow cytometry plots of tumor-infiltrating T_{conv} cells and T_{reg} cells. **(b)** Percentages of T_{reg} cells in distant tumors. **(c)** Calculated T_{conv}/T_{reg} ratio in distant tumors. Mean ± SEM is shown. Data represent cumulative results from seven independent experiments

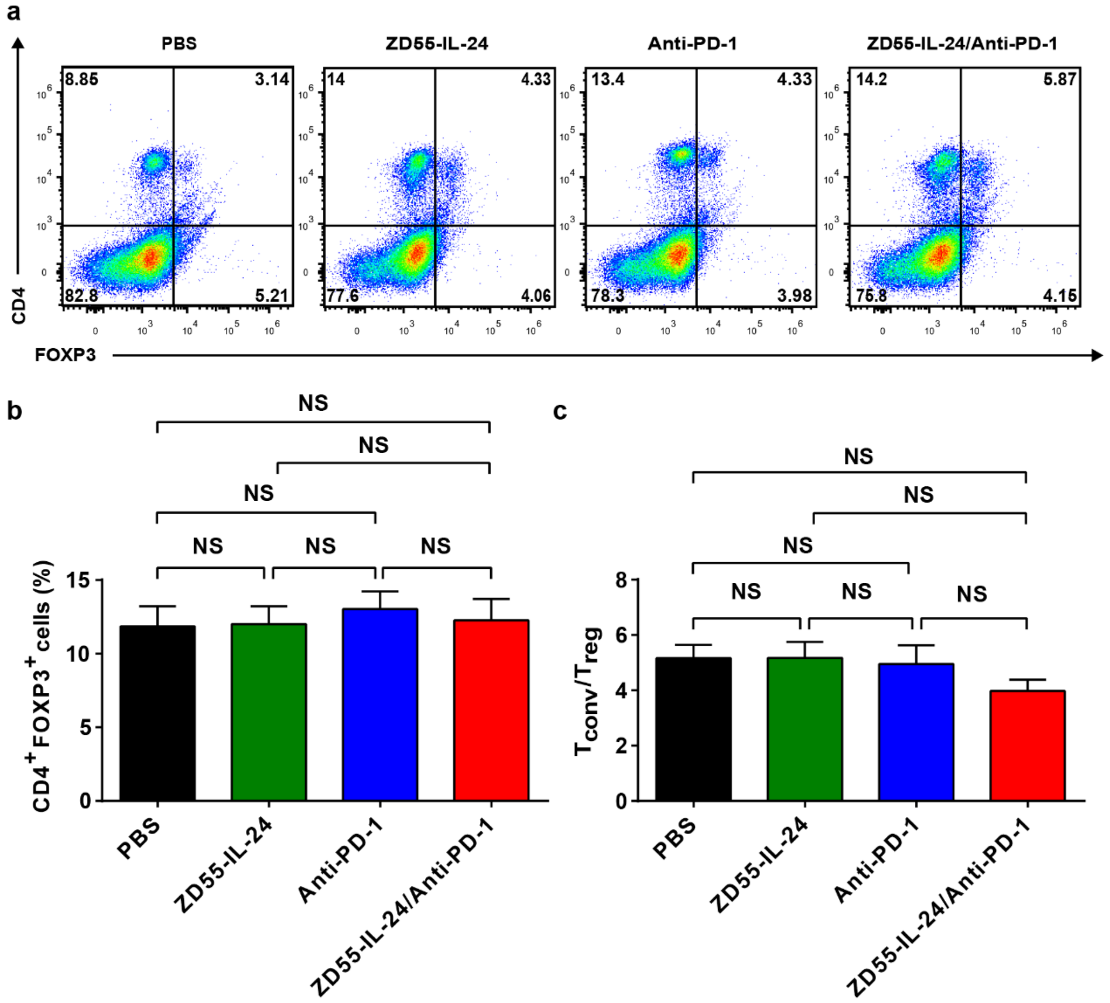

Supplementary Figure 3 ZD55-IL-24 can't help PD-1 blockade to further increase the T_{conv}/T_{reg} ratio in speens as well. C57BL/6 mice were treated as indicated in **Fig. 3a**, and then the spleen cells were isolated for flow cytometry analysis. **(a)** Representative flow cytometry plots of tumor-infiltrating T_{conv} cells and T_{reg} cells in spleens. **(b)** Percentages of T_{reg} cells in spleens. **(c)** Calculated T_{conv}/T_{reg} ratio in spleens. Mean ± SEM is shown. Data represent cumulative results from seven independent experiments
